# Severe musculoskeletal time-loss injuries and symptoms of common mental disorders in professional soccer: a longitudinal analysis of 12-month follow-up data

**DOI:** 10.1007/s00167-017-4644-1

**Published:** 2017-07-11

**Authors:** Ö. Kiliç, H. Aoki, E. Goedhart, M. Hägglund, G. M. M. J. Kerkhoffs, P. P. F. M. Kuijer, M. Waldén, V. Gouttebarge

**Affiliations:** 1 Academic Center for Evidence based Sports medicine (ACES), Academic Medical Center, Amsterdam, The Netherlands; 2 Department of Orthopaedic Surgery, Academic Medical Center, Amsterdam, The Netherlands; 3 St. Marianna University School of Medicine, Kawasaki, Japan; 4 Royal Netherlands Football Association (KNVB), FIFA Medical Center of Excellence, Zeist, The Netherlands; 5 Football Research Group, Linköping, Sweden; 6 Department of Medical and Health Sciences, Division of Physiotherapy, Linköping University, Linköping, Sweden; 7 Amsterdam Collaboration for Health and Safety in Sports (ACHSS), Academic Medical Center / VU Medical Center, Amsterdam, The Netherlands; 8 Coronel Institute of Occupational Health, Amsterdam Public Health Research Institute, Academic Medical Center, Amsterdam, The Netherlands; 9 Department of Medical and Health Sciences, Division of Community Medicine, Linköping University, Linköping, Sweden; 10 World Players’ Union (FIFPro), Scorpius 161, 2132 LR Hoofddorp, The Netherlands; 11 Division of Exercise Science and Sports Medicine, University of Cape Town, Cape Town, South Africa

**Keywords:** Common mental disorder, Professional football, Time-loss injury, Cohort study

## Abstract

**Purpose:**

Psychological factors have shown to be predictors of injury in professional football. However, it seems that this is a two-way relationship, as severe musculoskeletal time-loss injuries have shown to be associated with the onset of symptoms of common mental disorders (CMD). There is no longitudinal study performed exploring this interaction between symptoms of CMD and injuries. The purpose of this study was to explore the interaction between severe musculoskeletal time-loss injuries and symptoms of CMD in professional football players over a 12-month period.

**Methods:**

Players were recruited by their national players’ unions in five European countries. Symptoms of CMD included in the study were related to distress, anxiety/depression, sleep disturbance and adverse alcohol use.

**Results:**

A total of 384 professional football players were enrolled in the study, of whom 262 (68%) completed the 12-month follow-up period. The mean age of the participants at baseline was 27 ± 5 years, and they had played professional football for 8 ± 5 years on average. Symptoms of CMD at baseline were not associated with the onset of severe musculoskeletal time-loss injuries during the follow-up period with relative risks (and 95% CI) ranging from 0.6 (0.3–1.0) to 1.0 (0.5–2.2). In contrast, severe musculoskeletal time-loss injuries reported at baseline were associated with the onset of symptoms of CMD during the follow-up period with relative risks ranging from 1.8 (0.8–3.7) to 6.9 (4.0–11.9).

**Conclusion:**

No relationship was found between symptoms of CMD and the onset of severe musculoskeletal time-loss injuries. However, professional football players who suffered from severe musculoskeletal time-loss injuries are likely to develop subsequent symptoms of CMD. This study emphasizes the need for an interdisciplinary medical approach, which not only focuses on the physical but also on the mental health of professional football players. An early identification of players at risk of symptoms of CMD, such as those suffering from severe musculoskeletal injuries, creates the opportunity for an interdisciplinary clinical medical team to treat the players timely and adequately.

**Level of evidence:**

Prospective cohort study, Level II.

## Introduction

The overall risk of injury in professional football is estimated to be 1000 times higher when compared to typical high-risk industrial occupations like in manufacturing, construction or in the service sector [9]. In the UEFA Elite Club Injury Study during the seasons 2001–2008, a mean time-loss injury rate of 8.0 injuries per 1000 h was found, reaching up to 27.5 time-loss injuries per 1000 match hours. This study showed that typically a squad of 25 players could at least expect 50 injuries per season [12]. A 15-year epidemiological follow-up study among professional football players in Japan found that 2947 injuries occurred in 3984 matches and a mean annual injury rate of 21.8 per 1000 player hours [2]. Another 5-year prospective cohort study among professional football players competing at the Australian A-league presented a rate of time-loss injuries ranging from 58.9 to 109.7 time-loss injuries per squad of 25 players [19]. Time-loss injuries generally require medical treatment that can last from several days to several months, having a significant negative effect on the performance of the team [3, 21]. In addition, time-loss injuries that result in a long period without training or competition are considered as major adverse events for the career of a football player, leading even to early retirement in the worst case [15, 31, 34].

Several studies showed that not only physical but also psychological factors may influence the risk of a musculoskeletal injury [22–24, 28]. Psychological factors such as trait anxiety, negative-events-stress and daily hassle, were identified as predictors for injury in professional football [23]. While most of the studies are directed towards the incidence of musculoskeletal injuries, more attention has recently been given to the occurrence of symptoms of distress, anxiety/depression, sleep disturbance and substance abuse, typically referred to as common mental disorders (CMD), among professional football players. The prevalence of symptoms of CMD among European professional football players was found to extend to 32% for anxiety/depression, while the 12-month incidence ranged from 12% for distress to 37% for anxiety/depression [17]. Several studies showed that among others (e.g. career dissatisfaction, surgeries) severe time-loss injuries and life events were potential risk factors for symptoms of CMD [15, 16, 18, 20]. In 2015, cross-sectional analyses showed that professional football players who have sustained one or more severe musculoskeletal time-loss injuries during their career were two to nearly four times more likely to report symptoms of CMD than players who had not suffered from severe time-loss injury [15]. However, a longitudinal association between symptoms of CMD and severe time-loss injuries has not been established yet.

The present study aimed to explore the interaction between severe musculoskeletal time-loss injuries and symptoms of CMD in professional football players over a 12-month period. Two hypotheses were tested, namely that (I) professional football players reporting symptoms of CMD at baseline had an increased risk of severe musculoskeletal time-loss injury in the subsequent 12-month follow-up period and (II) professional football players suffering from severe musculoskeletal time-loss injuries at baseline were more likely to develop symptoms of CMD in the subsequent 12-month follow-up period.

## Materials and methods

This study was conducted in line with the STROBE (Strengthening the Reporting of Observational Studies in Epidemiology) statement for cohort studies [33]. The present study was an observational prospective cohort study with three measurements during follow-up, at baseline, at 6 months and at 12 months by means of questionnaires [33].

### Participants

The national players’ unions from five European countries were asked by the World Players’ Union (FIFPro) to assist in the recruitment of participants. The inclusion criteria were (I) being an active professional football player; (II) being 18 years or older; (III) being male; (IV) being a member of the national players’ union from Finland, France, Norway, Spain, or Sweden, which means committing significant time to football training and competing at the highest or second highest professional football level; and (V) being able to read and comprehend texts fluently in English, French or Spanish.

### Symptoms of common mental disorder

Symptoms of CMD included in the study were related to (1) distress, (2) anxiety/depression, (3) sleep disturbance and (4) adverse alcohol use. These symptoms of CMD were assessed using, respectively, the (1) Distress Screener, (2) 12-items General Health Questionnaire, (3) Patient-Reported Outcomes Measurement Information System (PROMIS) and (4) Alcohol Use Disorders Identification Test (AUDIT-C) [5, 7, 8, 14, 33, 36].

The Distress Screener (three items scored on a 3-point scale), which is based on the 4-dimensional symptom questionnaire (4DSQ) (e.g. “Did you recently suffer from worry?”), was used to measure distress in the previous 4 weeks (baseline) and in the previous 6 months (follow-up) [5, 33]. The 4DSQ, that is, Distress Screener in English, French and Spanish, has been validated for a recall period of up to several weeks [internal consistency: 0.6–0.7; test–retest coefficients: ≥0.9; criterion-related validity: area under receiver operating characteristic (ROC) curve ≥0.8] [5, 33]. A total score ranging from 0 to 6 was obtained by adding up the answers on the 3 items, a score of 4 or more indicating the presence of symptoms of distress [5, 33].

To assess symptoms of anxiety/depression in the previous 4 weeks (baseline) and in the previous 6 months (follow-up), the 12-items General Health Questionnaire (GHQ-12) was used (e.g. “Have you recently felt under strain?”) [14]. The GHQ-12 in English, French and Spanish has been validated for a recall period of up to several weeks (internal consistency: 0.7–0.9; criterion-related validity: sensitivity ≥0.7, specificity ≥0.7, area under ROC curve ≥0.8) [14, 30]. Based on the traditional scoring system, a total score ranging from 0 to 12 was calculated by adding up the answers on the 12 items, with a score of 3 or more indicating the presence of symptoms of anxiety/depression (area under ROC curve = 0.9) [14, 30].

Sleep disturbance in the previous 4 weeks (baseline) and in the previous 6 months (follow-up) was assessed through four single questions (e.g. “Have you recently had problems sleeping?”) scored on a 5-point scale (from “not at all” to “very much”) based on the Patient-Reported Outcomes Measurement Information System (PROMIS) [7, 36]. The PROMIS in English, French and Spanish has been validated for a recall period of up to several weeks (internal consistency: >0.9; construct validity: product–moment correlations ≥0.9) (for detailed information, see www.nihpromis.org). A total score ranging from 1 to 20 is obtained by summing up the answers to the four questions, a score of 13 or more indicating the presence of symptoms of sleep disturbance [7, 36].

To detect the level of alcohol consumption at present time (baseline) and in the previous 6 months (follow-up) the 3-items alcohol use disorders identification test was used (AUDIT-C) (e.g. “How many standard drinks containing alcohol do you have on a typical day?”) [8]. The AUDIT-C in English, French and Spanish has been validated for a recall period of up to several weeks (test–retest coefficients: 0.6–0.9; criterion-related validity: area under ROC curve = 0.7–0.9) [8, 27]. A total score ranging from 0 to 12 was obtained by adding up the answers on the three items, a score of 5 or more indicating the presence of adverse alcohol use [8].

### Severe musculoskeletal time-loss injuries

Football players were asked to report whether they had suffered from severe musculoskeletal time-loss injuries in the previous 4 weeks (baseline) and in the previous 6 months (follow-up). Severe musculoskeletal time-loss injury was defined as an injury that involved the musculoskeletal system, occurred during team activities (training or match), and led to either training or match absence for more than 28 days [13].

### Procedures

Based on the aforementioned variables included in the study, a baseline and two follow-up electronic questionnaires were arranged in English, French and Spanish (FluidSurveys™). The following descriptive variables were added: age, body height, body weight, duration of professional football career, level of play and team position. As several studies have shown that life events were associated with symptoms of CMD as well as with musculoskeletal injuries, the number of life events in the previous 6 months was also explored at baseline and follow-up with the validated Social Athletic Readjustment Rating Scale [6, 20, 24]. Each questionnaire took about 15–20 min to complete. The national players’ unions sent the information about the study per email to potential participants. Participants interested in the study gave their informed consent and were given access to the online questionnaire, which they were asked to complete within 2 weeks. At the end of the questionnaire, participants could leave their email address and give their informed consent for the follow-up online questionnaires. Follow-up questionnaires were sent per email 6 and 12 months later, with a request to complete them within 2 weeks. Reminders at baseline and follow-up were sent after 2 and 4 weeks. The responses to baseline and follow-up questionnaires were anonymized for reasons of privacy and confidentiality. Once completed, the electronic questionnaires were saved automatically on a secured electronic server that only the principal researcher could access. Players participated voluntarily in the study and did not receive any reward for their participation. This study is as part of a larger research project involving 11 countries for which ethical approval was obtained by the board of St Marianna University School of Medicine (April 16, 2014; Kawasaki, Japan) [16]. The present study was conducted in accordance with the Declaration of Helsinki (2013).

### Statistical analyses

The statistical software IBM SPSS 23.0 for Windows was used to perform all data analyses. Descriptive analyses (mean, standard deviation, frequency and range) were performed for all variables included in the study. An independent *T* test was used to explore whether loss to follow-up was selective by comparing baseline characteristics (all descriptive variables) of responders and non-responders [35].

In order to explore the interaction between independent (either symptoms of CMD or musculoskeletal time-loss injuries at baseline) and dependent (onset of either symptoms of CMD or musculoskeletal time-loss injuries during 12-month follow-up period) variables under study, three models were used: (1) unadjusted relative risk model, (2) relative risk model adjusted for age, and (3) relative risk model adjusted for age and life events, as both age and number of life events have been found to correlate with symptoms of CMD as well as with musculoskeletal injuries [1, 4, 20, 24]. All relative risk models took into account any new injuries or symptoms of CMD reported at the 6-month follow-up. We also assessed the interaction between two or more symptoms of CMD (comorbidity) and severe musculoskeletal time-loss injuries using the same aforementioned relative risk models (1–3). For the unadjusted model, a contingency table was used to calculate relative risks (RR). For both adjusted models, the Mantel–Haenszel risk ratio method was used to calculate the adjusted risk ratios [25]. For all three models, 95% confidence interval (CI) was calculated. Under the assumption that at least one out of ten players might suffer from a health condition under study, sample size calculation indicated that at least 138 participants were needed (confidence interval of 95%; precision of 5%) [35]. Expecting a response rate of approximately 40% (based on previous similar studies in professional football) and a loss to follow-up at 20%, we intended to invite at least 440 players [16, 18].

## Results

**Fig. 1:**
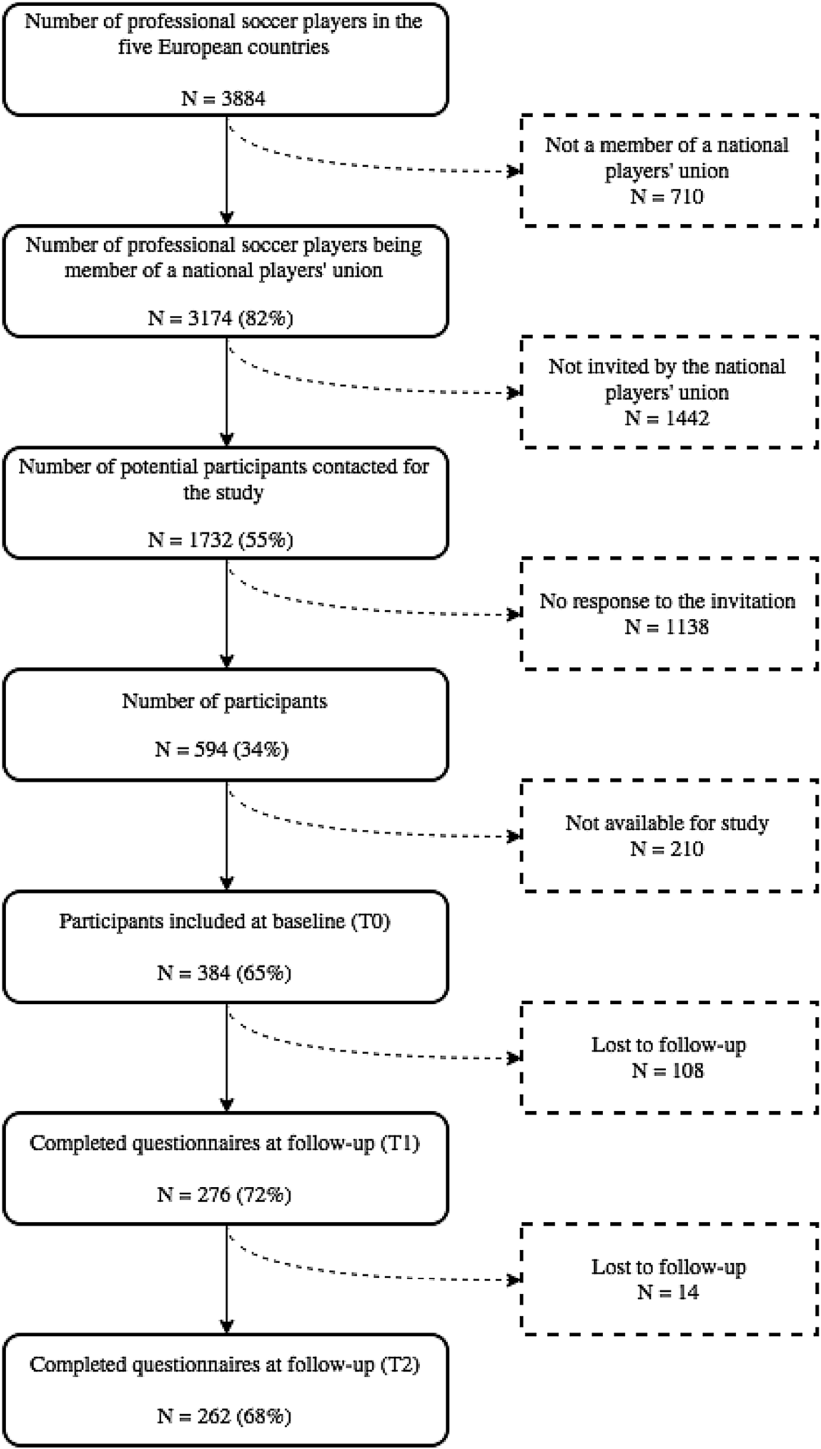
Flowchart of participants

Written informed consent to participate in the 12-month follow-up was given by 384 players (response rate of 65%). A total of 262 players completed the 12-month follow-up period (follow-up rate of 68%). The flowchart of the recruitment of the participants is presented in Fig. 1. The mean age of the 384 participants at baseline was 27 ± 5 years, and they had played professional football for 8 ± 5 years on average, of which 55% at the highest national level. From the 193 players that did not report any symptom of CMD at baseline, 37% reported a symptom of CMD in the subsequent 12 months. From the 336 players that did not report any severe musculoskeletal time-loss injury, 22% reported an injury in the subsequent 12 months. Main characteristics are presented in Table 1.

**Table 1:** Characteristics of the participants at baseline

	Total	No CMD	CMD	No MSD injury	MSD injury
Number of participants (%)	384	193 (50)	191 (50)	336 (88)	42 (11)
Age (years)	27 ± 5	28 ± 5	27 ± 4	27 ± 5	27 ± 4
Height (cm)	181 ± 7	182 ± 7	181 ± 7	182 ± 7	180 ± 7
Weight (kg)	78 ± 8	78 ± 8	77 ± 8	78 ± 8	76 ± 9
Duration of professional football career (years)	8 ± 5	9 ± 5	8 ± 4	8 ± 5	7 ± 4
Level of play (top league, %)	55	56	55	56	51
Field position (%)					
Goalkeeper	14	15	12	15	2
Defender	37	35	39	38	34
Midfielder	31	31	32	31	32
Forward	18	19	17	16	32
Educational level (%)					
No schooling completed	1	2	1	2	0
Nursery/elementary school	3	3	3	3	2
High school	51	50	52	51	51
Vocational/technical school	16	15	16	16	15
College, university, or equivalent	29	30	28	29	32
Recent life events (%)	61	60	62	61	68
Baseline prevalence (%)					
Distress	16			13	3
Anxiety/depression	32			25	6
Sleep disturbance	25			21	5
Adverse alcohol use	9			9	1
No MSD injury (%)		50	40		
MSD Injury (%)		3	8		

Values are mean ± SD unless otherwise stated

*CMD* common mental disorders, *MSD* musculoskeletal disorder

### Interactions between symptoms of CMD and severe musculoskeletal time-loss injuries

Symptoms of CMD at baseline were not associated with the risk of severe musculoskeletal time-loss injury during the 12-month follow-up period, with relative risks ranging from 0.6 (0.3–1.0) to 1.0 (0.5–2.2) for sleep disturbance and distress, respectively. All relative risks between symptoms of CMD at baseline and the risk of severe musculoskeletal time-loss injury during the subsequent 12-month are presented in Table 2.

**Table 2:** Relative risk (and 95% CI) between symptoms of CMD and onset of severe musculoskeletal time-loss injury

	Model (I): unadjusted	Model (II): adjusted for age	Model (III): adjusted for age and life events
Distress			
No	1.00	1.00	1.00
Yes	0.98 (0.55–1.74)	0.94 (0.43–1.99)	1.01 (0.45–2.24)
Anxiety/depression			
No	1.00	1.00	1.00
Yes	0.71 (0.43–1.19)	0.65 (0.35–1.11)	0.67 (0.35–1.13)
Sleep disturbance			
No	1.00	1.00	1.00
Yes	0.58 (0.32–1.04)	0.56 (0.25–1.00)	0.60 (0.30–1.31)
Adverse alcohol use			
No	1.00	1.00	1.00
Yes	0.59 (0.24–1.44)	0.60 (0.19–1.51)	0.59 (0.17–1.46)
≥2 symptoms of CMD			
No	1.00	1.00	1.00
Yes	0.64 (0.35–1.18)	0.61 (0.26–1.13)	0.62 (0.27–1.13)

*CMD* Common mental disorders, *CI* confidence of interval

Prevalence of severe musculoskeletal time-loss injuries at baseline was associated with symptoms of CMD during the 12-month follow-up period with relative risks ranging from 1.8 (0.8–3.7) to 6.9 (4.1–11.9) for adverse alcohol use and distress, respectively. These results show that professional football players who reported a severe time-loss injury at baseline are nearly 2–7 times more likely to develop symptoms of CMD in the subsequent 12 months by comparison with non-injured football players. All relative risks are presented in Table 3.

**Table 3:** Relative risk (and 95% CI) between severe musculoskeletal time-loss injuries and onset of symptoms of CMD

	Model (I): unadjusted	Model (II): adjusted for age	Model (III): adjusted for age and life events
Distress			
No MSD injury	1.00	1.00	1.00
MSD injury	6.90 (3.99–11.93)	6.31 (4.05–15.28)	6.01 (3.77–14.14)
Anxiety/depression			
No MSD injury	1.00	1.00	1.00
MSD injury	2.91 (2.27–3.74)	2.89 (3.35–167.65)	2.90 (3.43–113.23)
Sleep disturbance			
No MSD Injury	1.00	1.00	1.00
MSD injury	4.22 (2.64–6.75)	4.10 (2.93–13.86)	4.01 (2.81–13.74)
Adverse alcohol use			
No MSD injury	1.00	1.00	1.00
MSD injury	1.94 (0.88–4.26)	1.99 (0.92–3.76)	1.82 (0.84–3.67)
≥2 symptoms of CMD			
No MSD injury	1.00	1.00	1.00
MSD injury	5.84 (4.26–8.01)	5.65 (–)^a^	5.40 (–)^a^

*CMD* Common mental disorder, *MSD* musculoskeletal disorder, *CI* confidence of interval

^a^ No case in some subsamples and thus impossible to calculate 95% CI

## Discussion

The most important finding of the present study was that professional football players who suffered from severe musculoskeletal time-loss injuries are likely to develop subsequent symptoms of CMD. Contrary to our hypothesis, no relationship was found between symptoms of CMD and the onset of severe musculoskeletal time-loss injuries during a subsequent 12-month follow-up period among professional football players. We do acknowledge a potential power problem with some of the 95% confidence intervals just barely overlapping the value 1.0. With regard to these results, we may assume that self-reported symptoms of CMD as assessed in our study might not be as severe as expected because those did not cause severe musculoskeletal time-loss injuries among participants. An assumption is that clinically diagnosed CMD, which is more severe than self-reported symptoms of CMD, might be more likely to induce severe time-loss injuries among football players. As previously mentioned, some studies found an association between psychological factors such as trait anxiety, negative-events-stress and daily hassle and the occurrence of injuries [23]. As life events have shown to be predictors of injury, it is most likely that these life events cause stress that reduces attention and mental performance that consequently modifies the reaction time of the athlete in situations with a possible risk of injury [10, 11]. For instance, poor reaction time was found to be a predictor of injury in a previous study on amateur football players [11]. Also there is evidence that athletes show more pronounced readiness to take risks due to factors such as insufficient caution, adventurous spirit or higher outward expression of anger (more foul play) [10, 24]. One might logically assume that if mental performance seems to be associated with injury, symptoms of CMD should also be significantly associated with these injuries. An explanation why this association was not present in our results is that we assessed the occurrence of severe musculoskeletal time-loss injuries that lead to a layoff period of more than 4 week. One might hypothesize that symptoms of CMD as reported in our study might lead to less severe musculoskeletal injuries. This should be subject to further investigations. Regardless these results, the majority of the current and retired professional football players report that symptoms of CMD influence football performances, which is along with injuries a major reason to monitor the occurrence of symptoms of CMD [32].

In contrast to the association between symptoms of CMD and the onset of severe musculoskeletal time-loss injuries, severely injured professional football players were found to be nearly 2–7 times more likely to develop symptoms of CMD in the subsequent 12 months by comparison with non-injured football players. This longitudinal association was significant for all symptoms of CMD under study, confirming previous cross-sectional analyses conducted with the same study population [15]. It is worth mentioning that less severe musculoskeletal time-loss injuries logically are expected to have less psychological impact and might not be associated with the onset of symptoms of CMD as strong as severe musculoskeletal time-loss injuries. Also severe musculoskeletal time-loss injuries are a major adverse life event for professional football players [13, 15, 31]. In addition, studies among other populations have proven that adverse life events have a causal relationship with symptoms of CMD [15, 16, 18, 20]. With this study, severe musculoskeletal time-loss injuries can be considered as major adverse life events for professional football players that are likely to cause symptoms of CMD.

A potential limitation of the present study might be that the data was self-reported as the questionnaires were answered by professional football players themselves. Measurement through medical professionals might have led to less subjective information and additional information with regard to the number of days until Return To Play. Another limitation could be the response and follow-up rates, namely 65 and 68%, respectively. Epidemiologists have suggested acceptable follow-up rates ranging from adequate to very good or required with a follow-up rate of, respectively, 50, 70 and 80% [26]. Despite that we strived to reach a follow-up rate of at least 80%, the 68% achieved in our study seems to be good compared with the aforementioned suggested acceptable follow-up rates. Also a monthly survey over the follow-up period might have given more valid data than the 6-month period used in this study. However, it is well know that professional athletes, especially football players, remain reluctant to complete surveys repeatedly. Although the native language of participants from Finland, Norway and Sweden was not administered in the scales used to measure symptoms of CMD, we feel that this has no negative effect on the validity of the collected data because an inclusion criterion was that the participants were able to read and comprehend texts fluently in English, French or Spanish, and secondly most members of the players’ unions from Finland, Norway and Sweden are studying at an English academy arranged by their players’ union. Also the baseline measurements vary between the leagues (with Nordic countries having Spring-Fall season, and France and Spain having Fall-Spring season). It is worth mentioning that only male football players are analysed and outcomes could differ among female professional football players. An important strength is the longitudinal design of this study among nearly 400 professional football players concerning a sensitive topic like mental health that remains taboo even today. This is, to the authors’ knowledge, the first prospective cohort study exploring this interaction. This longitudinal study design allows the establishment of a causal exploration between symptoms of CMD and severe musculoskeletal time-loss injuries.

Musculoskeletal injuries have a negative impact on the performance of a player and his team and consequently, the possible association between symptoms of CMD and the onset of less severe musculoskeletal time-loss injuries should be explored [3, 21]. If the abovementioned possible association might be present, it is important to acknowledge and recognize these symptoms of CMD and treat them in order to minimize the risk of or prevent a musculoskeletal time-loss injury. This study emphasizes the importance of the awareness, acknowledgement and recognition of symptoms of CMD. According to the results of this study, one can assume that a player that suffers from a severe musculoskeletal time-loss injury will be likely to develop symptoms of CMD. One can logically assume that these symptoms of CMD might have consequences for their performance and quality of life as mentioned in a previous study [32].

The clinical relevance of this study is that it emphasizes the need for an interdisciplinary medical approach, which not only focuses on the physical but also on the mental health of football players. Not only physical but also psychological readiness has shown to increase athletes’ perceived likelihood of a successful return to play [29]. Consequently, an early identification of players at risk of symptoms of CMD creates the opportunity for an interdisciplinary medical team to recognize these symptoms timely and treat players in an early stage in order to prevent these symptoms getting worse and in order to remain or improve their performance and quality of life. One can logically assume that this may lead to a faster as well as a safer return to play.

## Conclusion

No relationship was found between symptoms of CMD and the onset of severe musculoskeletal time-loss injuries during a subsequent 12-month follow-up period among professional football players. However, severely injured professional football players were found to be nearly 2–7 times more likely to develop symptoms of CMD in the subsequent 12 months by comparison with non-injured football players. An early identification of players at risk of symptoms of CMD, such as those suffering severe musculoskeletal injuries, creates the opportunity for an interdisciplinary medical team to treat the players timely and adequately.
